# Innovative Strategies for Technical-Economical Optimization of FDM Production

**DOI:** 10.3390/polym15183787

**Published:** 2023-09-16

**Authors:** Dragoș Gabriel Zisopol, Maria Tănase, Alexandra Ileana Portoacă

**Affiliations:** Mechanical Engineering Department, Petroleum-Gas University of Ploiești, 100680 Ploiesti, Romania; zisopold@upg-ploiesti.ro

**Keywords:** 3D printing, value analysis, optimization, printing parameters, PLA, ABS, annealing, mechanical properties

## Abstract

This article introduces a multi-objective optimization approach for determining the best 3D printing parameters (layer thickness and infill percentage) to efficiently produce PLA and ABS parts, extensively analyzing mechanical behavior under tests for different traits such as tensile strength, compression, flexural, impact, and hardness. The value analysis method is used to optimize settings that balance use value (*V_i_*- represented by mechanical characteristics) and production cost (*C_p_*). Findings reveal that the infill percentage significantly influences the *V_i_/C_p_* ratio for tensile, compression, and hardness tests, while flexural tests are influenced by layer thickness. Impact strength is influenced nearly equally by both factors, with material-specific variations. The desirability function proved useful for optimizing processes with multiple responses, identifying the optimal parameters for the FDM process: a layer thickness of 0.15 mm with 100% infill percentage for PLA, a layer thickness of 0.20 mm with 100% infill percentage for annealed PLA, and a layer thickness of 0.15 mm with 100% infill percentage for ABS. Overall, this study guides efficient 3D printing parameter selection through a technical-economic optimization based on value analysis.

## 1. Introduction

In the contemporary landscape of additive manufacturing, fused deposition modeling (FDM) has emerged as a prominent technique, offering novel avenues for design innovation and production efficiency. Out of all the available 3D printing methods, FDM stands out as an economical and fast printing technique [1]. However, the optimization of FDM extends beyond technical precision, encompassing economic considerations that underpin sustainable manufacturing practices. The effectiveness of FDM is influenced by a range of process parameters, which can significantly affect both the cost and quality of the 3D-printed components [2,3].

While FDM’s capacity for intricate design realization is widely acknowledged, its optimal implementation necessitates a holistic understanding of the multifaceted parameters governing the manufacturing workflow. Moreover, the intricate amalgamation of material utilization, production duration, and mechanical performance within the FDM framework underscores the significance of pioneering strategies that harmonize technical finesse with cost-conscious practices. The efficacy of 3D printing, alongside the mechanical characteristics of the end component, hinge on a spectrum of printing parameters including layer thickness, printing speed, infill density, filling pattern, printing material, and various other factors [4]. The predefined printing process parameters provided by manufacturers are not guaranteed to produce high-quality printed products, as numerous variables can influence the printing procedure. Incorrectly configured initial printing parameters can lead to prolonged processing times, avoidable resource consumption, and diminished tensile strength, and consequently inflate production expenses, generate material wastage, and pose challenges for end-users [5,6,7,8,9,10]. In recent times, a substantial body of research literature has emerged focused on improving the setup of these parameters.

The study detailed in [4] investigated how different printing settings affect the strength of 3D-printed objects. Five key parameters, layer thickness, printing speed, infill density, filling pattern, and printing material, were analyzed using a design of experiment (DOE) approach. Multi-objective optimization was applied to enhance mechanical properties while minimizing time and material usage. The results demonstrated significant improvements, reducing printing time by 72.39% and increasing mass by 9.06% while enhancing mechanical performance.

Maguluri et al. [11] explored the effects of three crucial printing factors—infill density, extrusion temperature, and printing speed—on the hardness of poly-lactic acid (PLA) components. The Taguchi design of experiment (DOE) approach was applied to efficiently assess the printing settings that enhance the hardness of the printed parts while minimizing the number of experiments conducted. The analysis employed signal-to-noise (S/N) ratios to identify the optimal parameters, and the relative contributions of these factors were quantified using analysis of variance (ANOVA). The findings underscore the significant influence of extrusion temperature on the hardness of 3D-printed PLA samples, whereas printing speeds exhibit a comparably minor impact on this property.

The research in [12] investigated the impact of different printing parameters (layer thickness, build orientation, and raster angle) on the mechanical properties of PLA specimens, using a design of experiment (DOE) approach. The findings reveal a negative correlation between layer thickness and ultimate tensile strength (UTS). Higher layer thickness leads to a decrease in mean UTS. A build orientation of 45 degrees tends to exhibit the highest tensile strength, while the raster angle has a less significant impact compared to the other two process parameters. The maximum observed UTS was 46.65 MPa for a layer thickness of 0.1 mm, a raster angle of 30 degrees, and a build orientation of 0 degrees.

In [13], the application of a multi-objective optimization was explored to refine the process parameters (infill patterns, infill percentage, printing speed, and layer thickness) for FDM 3D printing of PLA components, evaluating surface roughness, printing time, and filament length consumed as response variables.

A similar study was performed in [14], with the aim being to examine the impact of certain input experimental factors, specifically infill density, layer thickness, and support style, on response parameters such as the build time of a part and surface roughness for an acrylonitrile butadiene styrene (ABS) polymer using Taguchi design.

Vishwas et al. [15] focused on assessing how process parameters (model orientation, layer thickness, and shell thickness) in FDM influence key characteristics like ultimate tensile strength and the dimensional accuracy of ABS and nylon materials. Based on Taguchi analysis, it was highlighted that orientation angle and shell thickness notably impact ultimate tensile strength and moderately influence dimensional accuracy, with a focus on achieving optimal manufacturing outcomes. Similarly, in [16], the ANOVA analysis emphasized the nozzle diameter as the significant factor for a PLA 3D-printed part’s hardness, while for the tensile strength, the significant factor was identified to be the printing direction.

Many other studies [5,17,18,19] have focused on establishing the optimal setting for printing parameters to enhance the mechanical characteristics of 3D-printed parts.

Sustainable manufacturing aims to integrate quality, environmental consequences, and cost implications, leading to a shift in the focus of optimization objectives [20]. Therefore, technical–economical optimization must be considered when analyzing the efficiency of FDM process. In this sense, using value analysis to assess the mechanical characteristics of 3D-printed parts is an important and interesting effort in modern manufacturing. As the adoption of additive manufacturing expands across various industrial sectors, ensuring the optimal performance and cost-effectiveness of fabricated parts becomes increasingly imperative. The studies in [21,22,23,24,25,26] have investigated the cost-effectiveness and the environmental impact of additive manufacturing.

Refocusing on the relationship between production costs and printed component production, the current emphasis [21] lies on optimizing energy efficiency and reducing expenses in the manufacturing process, particularly in material-extrusion additive manufacturing. This is crucial for achieving sustainability and cost-effectiveness in production. Notably, MEX 3D printing consistently delivers high-quality parts, especially when employing costly high-performance polymers, which find applications in the biomedical, automotive, and aerospace sectors. In this context, [21] delves into the examination of two vital parameters, namely energy consumption, which can be translated into production costs and tensile strength.

The study in [22] used statistical modeling tools to assess various metrics related to compression and energy consumption in 3D printing. These metrics included printing time, weight, printing energy consumption, specific printing energy, specific printing power, compression strength, compression modulus of elasticity, and toughness. Among the factors examined, the layer thickness emerged as the most significant control parameter, while nozzle temperature and raster deposition angle had less impact on the outcomes.

The work detailed in [23] extensively explored the influence of seven universal and machine-agnostic 3D-printing configurations on both the energy usage and mechanical properties of parts made from PLA using the MEX 3D-printing method. The results showed that the printing speed and layer thickness had the most significant impact on energy consumption in the study. Additionally, the infill density and orientation angle were identified as the primary factors affecting compressive strength.

The investigation performed in [26] found that layer thickness and infill density are the first and the second most important factors in energy consumption, respectively, when manufacturing 3D-printed parts.

In specialized works, both the term “Value Engineering” and “Value Analysis” are used, both reflecting the same content but at different stages of the product’s existence [27]. Value engineering/analysis represents a method of systematic and creative research and design that, through a functional approach, aims to design and achieve the functions of the studied object with minimal expense, in quality conditions that meet the users’ needs, in line with socio-economic requirements [27].

Starting from social needs and based on the latest advancements in science and technology, value engineering/analysis studies aim to establish an optimal relationship between the use value of the analyzed product (*V_i_*) and the direct and indirect production costs it generates (*C_p_*).

The fundamental relationship that validates the design of a product in the presented context is [27]:

(1) $$\frac{\;V_{i}}{C_{p}}\to \max$$

where *V_i_* is measured in performance units and *C_p_* is expressed in monetary units.

The value of the *V_i_/C_p_* ratio can be increased through [27]:

✓ Increasing product performance while maintaining a constant price;
✓ Reducing the price while maintaining constant performance;
✓ Increasing performance more than cost;
✓ Increasing performance levels while reducing costs;
✓ Reducing performance, but with an even greater reduction in cost.

This study brings a novel perspective to this area by applying the value-analysis concept to enhance the mechanical characteristics of 3D-printed parts through the optimization of process parameters. In the field of additive manufacturing research, where the pursuit of optimal performance and cost-efficiency is essential, this approach stands out for its comprehensive consideration of multiple factors. Existing literature often focuses on isolated aspects of 3D printing, such as material properties or specific process parameters. This study bridges the gap by encompassing a comprehensive evaluation that investigates mechanical behavior established based on experimental testing (tensile, compression, bending, resilience, hardness) in correlation with the production cost, both for as-built and for annealed specimens, made from different materials (PLA and ABS).

## 2. Materials and Methods

The printing parameters affecting the mechanical properties and production cost of 3D-printed samples are the layer thickness (0.10 mm/0.15 mm/0.20 mm) and infill percentage (50%/75%/100%). The mechanical properties investigated were previously determined by the authors of the present work, namely tensile strength [6,9,28], compression strength [8], flexural strength [7,9], impact strength [29], and hardness [28,30].

A Raise E2 3D printer (Irvine, CA, USA), having a volume capacity of 330 × 240 × 240 mm, was used for the printing process.

The specific printing options used in the mentioned studies (Table 1) were: build orientation X-Y, model lines, and 45° orientation.

Table 2 presents the provider characteristics extracted from data sheets for the PLA and ABS filaments used in the investigation. ABS and PLA filaments were supplied by Polymaker (Utrecht, The Netherlands).

For the annealing heat treatment, the samples were kept for a period of 3 h at a temperature of 75 °C, with slow cooling in an oven.

For each investigated mechanical property, a design of experiments (DOE) full factorial design method was used through Minitab 19 software to optimize the ratio between the use value and production cost of PLA and ABS 3D-printed samples. For each printing parameter, three levels were considered, as can be seen in Table 3.

The total number of experiments required is determined by the function of the number of input factors (n) and the number of levels (k). This resulted, therefore, in an orthogonal array of 3^2^ values (see Table 4).

To calculate the production cost, we used the relation [27]:

(2) $$C_{p}=C_{mat}+C_{e}$$

(3) $$C_{mat}=Q_{mat}\times P_{m}$$

(4) $$C_{e}=T_{e}\times C_{en}\times P_{en}$$

where: $C_{p}$ is the production cost [Euro], *C_mat_* is the cost of material [Euro], *C_e_* is the cost of energy [Euro], $Q_{mat}$—material consumption [g] for a single sample, ${\;P}_{m}$—price of material [Euro/g], $T_{e}$—printing time [h], $C_{en}$—energy consumption [kW], $P_{en}$—the price of electricity [Euro/kWh].

For the annealed samples, the production cost *C_p_^a^* was calculated with the formula:

(5) $$C_{p^{a}}=C_{p}+\left(T_{a}\times C_{{en}^{a}}\times P_{en}\right)/n_{s}$$

where *C_p_* is the production cost calculated with Formula (1) for the as-built samples, *T_a_* is the annealing time [h], *C_en_^a^* is the energy consumption of the annealing device [kW], and *n_s_* is the number of samples annealed simultaneously in the oven.

In the calculations, the following values were considered: *C_en_* = 0.35 kW (the energy consumption of the Raise E2 3D printer), *P_m_* = 0.022 Euro/g (for PLA [31]) and *P_m_* = 0.021 Euro/g (for ABS [31]), *P_en_* = 0.28 Euro/kWh [32], *T_a_* = 3 h, *C_en_^a^* = 1 kW, *n_s_* = 45. The material consumption and printing time for each 3D-printed sample was taken from the software of the 3D printer, and the energy consumption for the 3D-printing process and for annealing, respectively, were taken from the devices’ specifications.

Testing conditions, such as the shape and dimension of the samples for each experimental investigation performed, are presented in Table 5.

## 3. Results and Discussion

### 3.1. Application of Value Analysis for Analyzing the Mechanical Behavior of ABS and PLA 3D-Printed Materials

#### 3.1.1. Tensile Testing

Table 6 and Table 7 show the results of calculations performed using relations (2…5), to determine the production cost for PLA (as-built and annealed PLA A) and ABS samples, respectively, used for tensile testing.

Similar to their impact on mechanical properties, the 3D-printing parameters were observed to significantly influence the production cost due to energy consumption [25].

**Figure 1:**
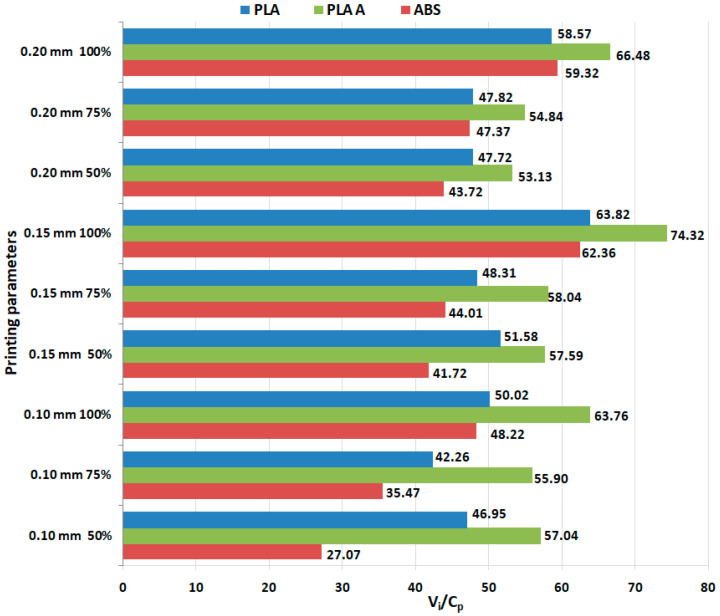
Ratios between use values and production costs in the case of tensile testing of 3D-printed materials.

Figure 1 illustrates a visual representation that highlights the ratios between use values (ultimate tensile strength) and production costs, based on the results obtained from tensile testing conducted on 3D-printed PLA and ABS materials.

Although the heat treatment adds supplementary costs to the production, the results from Figure 1 shows that, in all analyzed cases, the annealed PLA consistently exhibits the highest ratios, because of its improved mechanical performance, followed by as-built PLA and then ABS, indicating that annealed PLA might provide the best balance between functional performance and production cost, in the case of tensile characteristics. The highest ratio is obtained for 0.15 mm layer thickness and 100% infill percentage for all the considered materials.

The data from Table 8 indicate that, in the case of tensile specimens, the ratio *V_i_/C_p_* increased from 11.65% to 32.29%, based on increasing performance more than cost (the production cost of annealed specimens increased from 2.79% to 3.74%, but the ultimate tensile strength increased from 15.80% to 36.25%).

**Figure 2:**
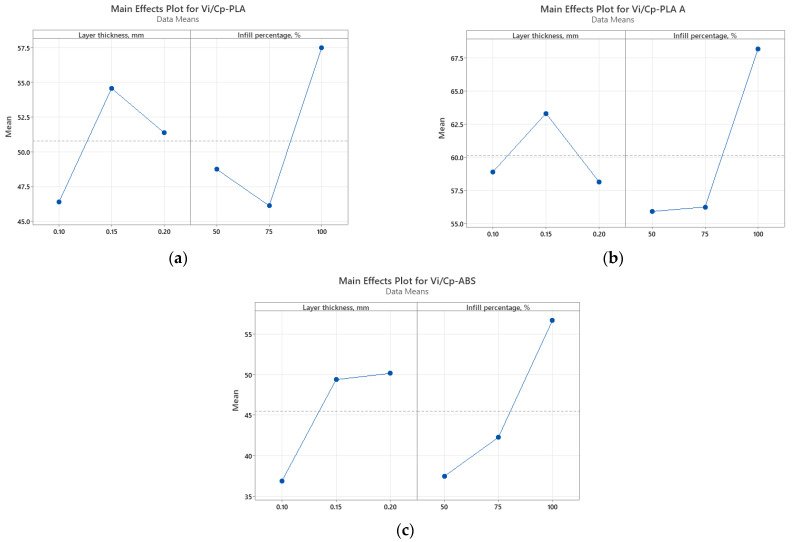
Main effect plots for tensile strength: (**a**) PLA; (**b**) PLA A; (**c**) ABS.

**Figure 3:**
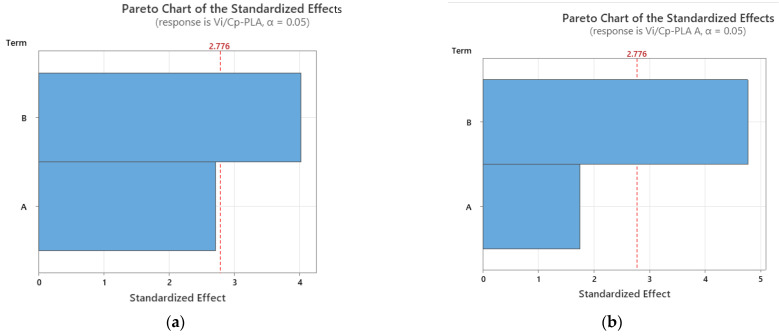

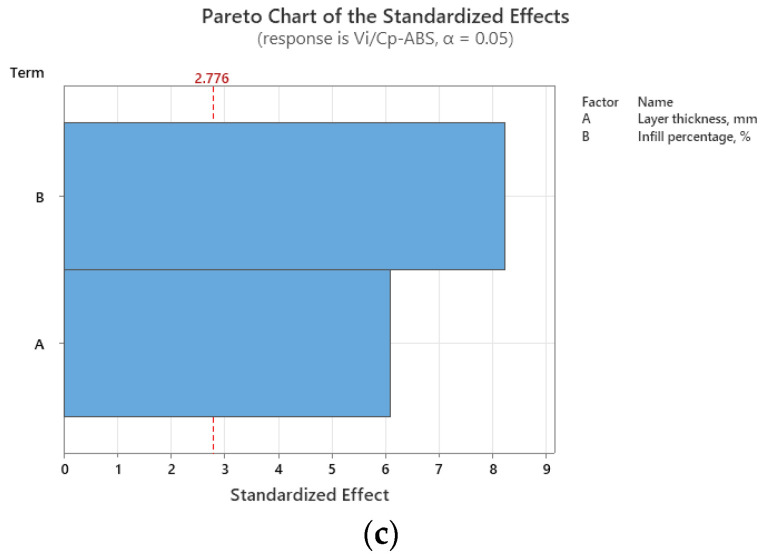
Pareto charts for tensile strength: (**a**) PLA; (**b**) PLA A; (**c**) ABS.

It was found from Figure 2 that for all analyzed materials, the highest *V_i_/C_p_* ratio was obtained at 100% infill percentage and 0.15 mm layer thickness (for as-built and annealed PLA) and 0.20 mm (for ABS, where it can also be observed that the ratio *V_i_/C_p_* increased with the increase in infill percentage). The ANOVA analysis (performed with Minitab 19 software and setting the *p*-value at the conventional threshold of 0.05) revealed that the statistically significant factor influencing the ratio *V_i_/C_p_* for tensile strength is the infill percentage. The same conclusion can be drawn from Pareto charts presented in Figure 3.

In contrast to the findings reported in [24], where layer thickness exhibited the primary influence on energy consumption, followed by infill density, the current study identifies that the ratio *V_i_*/*C_p_* is notably more influenced by variations in infill percentage.

#### 3.1.2. Compression Testing

Table 9 and Table 10 show the results of calculations performed using relations (2…5), to determine the production cost for PLA and ABS samples, respectively, used for compression testing.

Compared with [22], where energy consumption was evaluated and it was found that the energy is drastically reduced with an increase in layer thickness, in Table 10 the same trend is found in the costs calculated.

**Figure 4:**
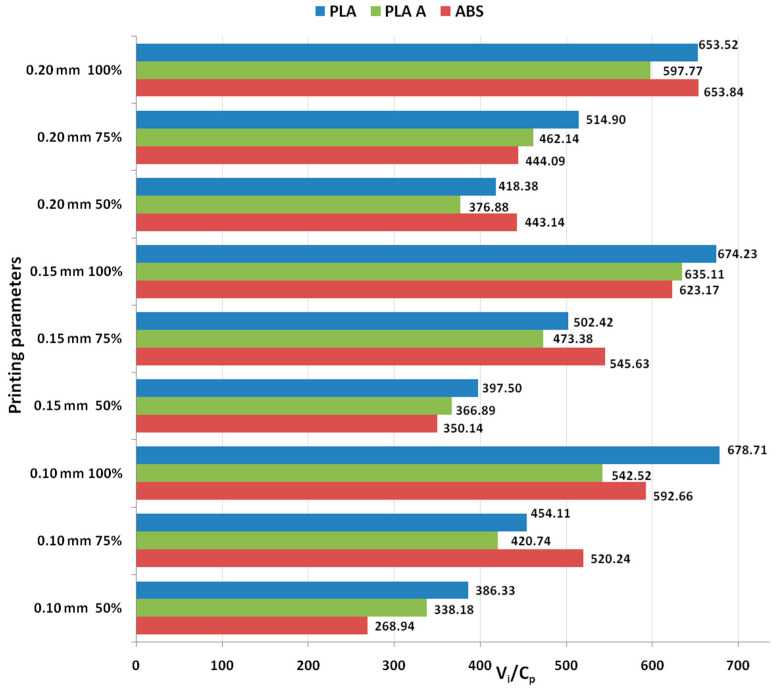
Ratios between use values and production costs in the case of compression testing of 3D-printed materials.

Figure 4 represents a graphical image showcasing the ratios between use values (compressive stress) and production costs, specifically focusing on the outcomes of compression testing for PLA and ABS 3D-printed materials.

The data in Table 11 reveals that, when constructing compression specimens, the ratio of *V_i_* (performance) to *C_p_* (production cost) decreased by 6.14% to 25.1%. This decrease occurred because the increase in performance was less pronounced than the rise in production costs. Specifically, for annealed specimens, production costs increased between 17.74% and 25.52%, while compressive stress increased by 5.78% to 13.84%. Notably, the only exception was observed in the case of specimens with a 0.1 mm layer thickness and 100% infill percentage, where the compressive stress was 5.88% lower for annealed specimens compared to the as-built ones.

**Figure 5:**
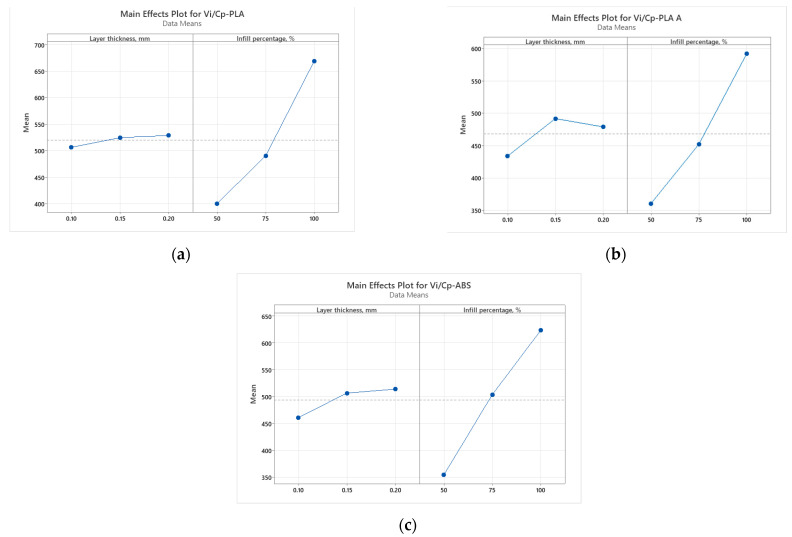
Main effect plots for compression strength: (**a**) PLA; (**b**) PLA A; (**c**) ABS.

**Figure 6:**
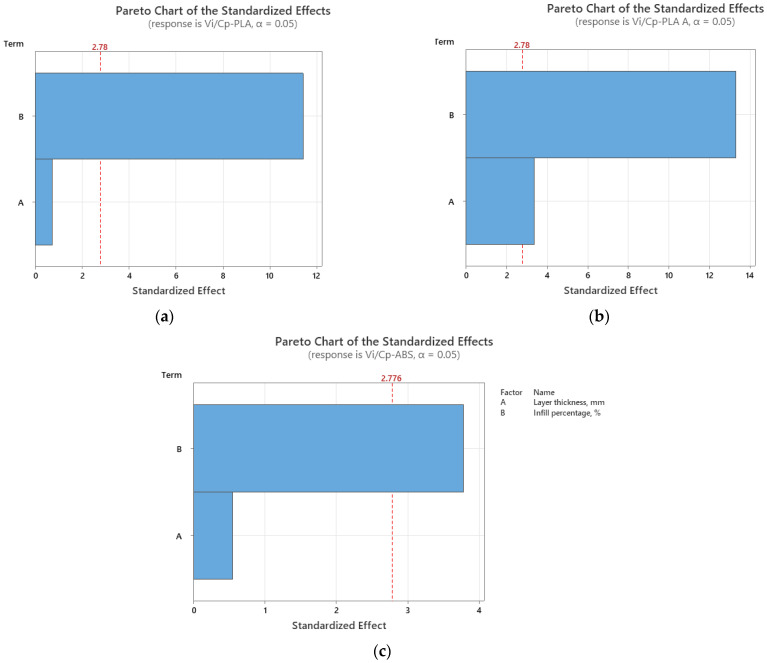
Pareto charts for compression strength: (**a**) PLA; (**b**) PLA A; (**c**) ABS.

The graphs from Figure 5 show that for all analyzed materials, the highest *V_i_/C_p_* ratio was obtained at 100% infill percentage and 0.20 mm layer thickness (for as-built PLA and ABS) and 0.15 mm (for annealed PLA). Also, for all analyzed materials, the ratio *V_i_/C_p_* increased with the increase in infill percentage. The ANOVA analysis and Pareto charts (Figure 6) reveal that the statistically significant factor influencing the *V_i_/C_p_* ratio for compression strength is the infill percentage. The same observation was formulated in [26], where it was found that, in terms of compressive strength, the infill density emerged as the primary influencing factor.

#### 3.1.3. Flexural Strength Testing

Table 12 and Table 13 show the results of calculations performed using relations (2…5), to determine the production cost for PLA and ABS samples, respectively, used for flexural strength testing.

**Figure 7:**
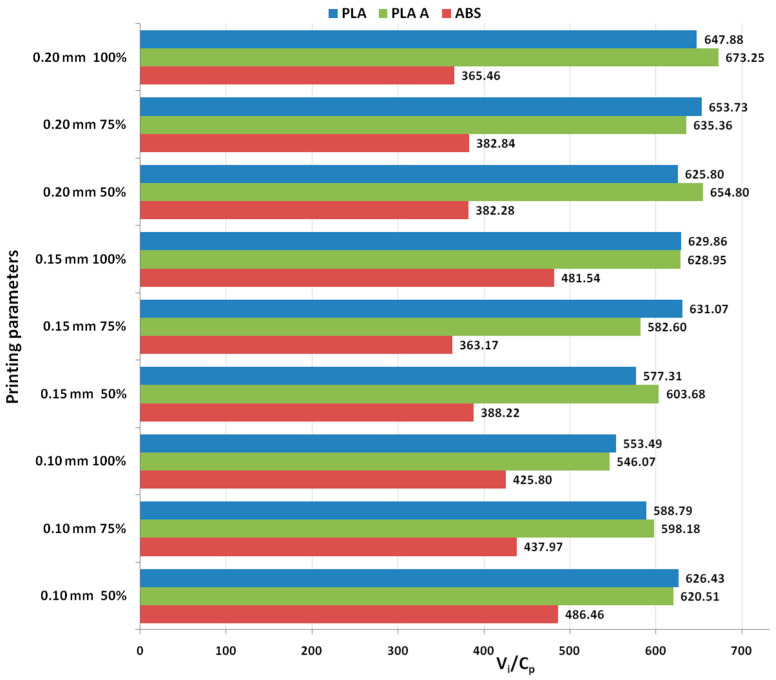
Ratios between use values and production costs in the case of flexural testing of 3D-printed materials.

Figure 7 provides a comparative visualization of the ratios between use values (flexural strength) and production costs, specifically in the context of flexural testing for PLA and ABS 3D-printed materials.

The obtained results indicating the notable influence of the 3D-printing configurations on the flexural strength of 3D-printed components are in good agreement with the observations from [25].

The information extracted from Table 14 reveals that in the context of flexural specimens, the ratio *V_i_/C_p_* shows remarkable similarity between annealed and as-built specimens. This similarity is demonstrated by minimal fluctuations, with slight increases ranging from 1.59% to 4.63%, or decreases spanning from 0.95% to 7.68%. This phenomenon arises because the increase in flexural strength for annealed samples closely parallels the corresponding rise in their production costs. Essentially, the incremental cost of production for annealed specimens corresponds closely with their enhanced flexural strength, resulting in a finely balanced *V_i_/C_p_* ratio between the two specimen types.

**Figure 8:**
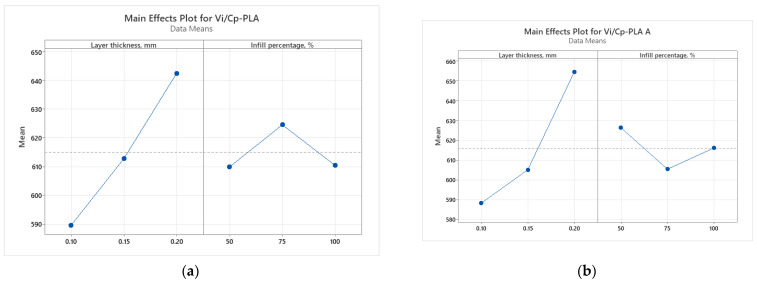

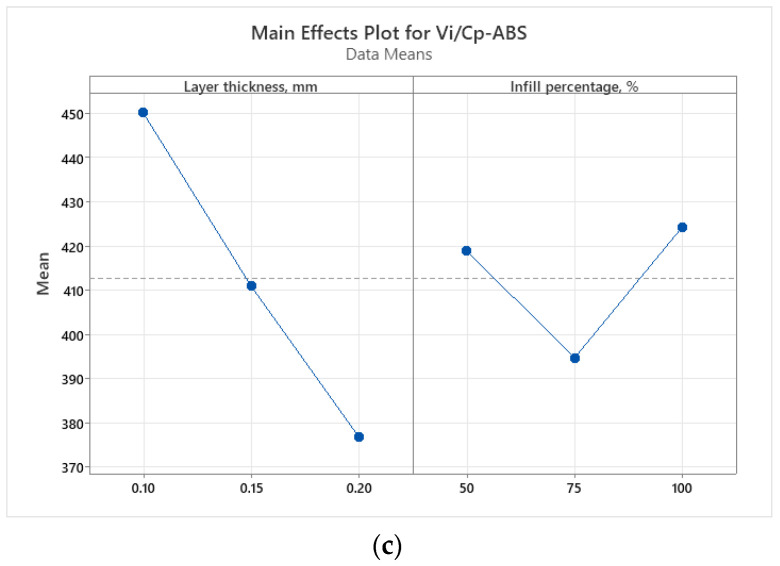
Main effect plots for flexural strength: (**a**) PLA; (**b**) PLA A; (**c**) ABS.

**Figure 9:**
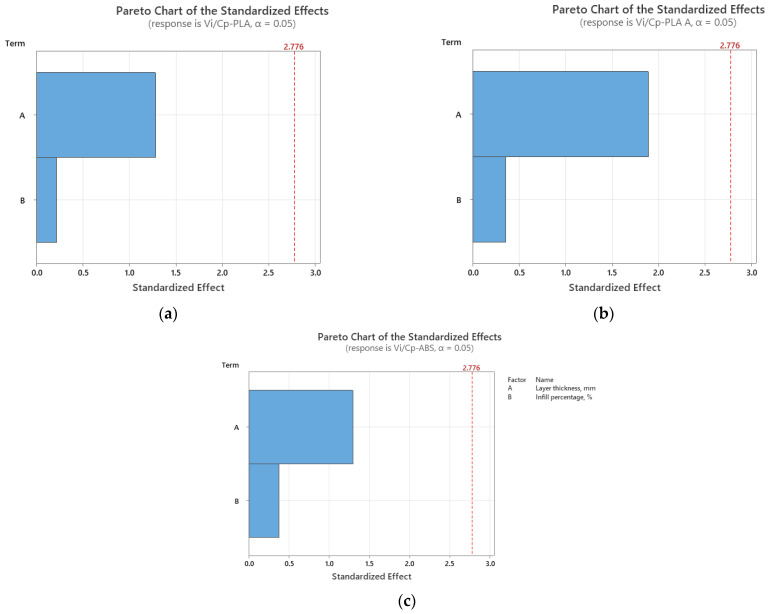
Pareto charts for flexural strength: (**a**) PLA; (**b**) PLA A; (**c**) ABS.

Analyzing the graphs shown in Figure 8, it can be observed that the highest ratio *V_i_/C_p_* was obtained at 0.20 mm layer thickness (for as-built and annealed PLA, where the ratio *V_i_/C_p_* increased with the increase in layer thickness) and at 0.20 mm layer thickness (for ABS, where the ratio *V_i_/C_p_* decreased with the increase in layer thickness). The ANOVA analysis and Pareto charts (Figure 9) revealed that the statistically significant factor influencing the ratio *V_i_/C_p_* for flexural strength is the layer thickness.

#### 3.1.4. Impact Testing

Table 15 and Table 16 show the results of calculations performed using relations (2…5) to determine the production cost for PLA and ABS samples, respectively, used for impact testing.

**Figure 10:**
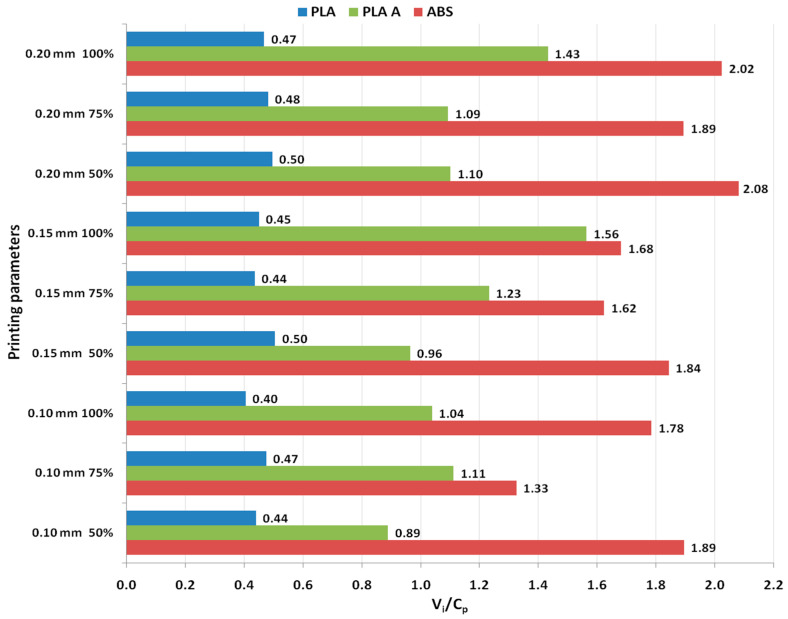
Ratios between use values and production costs in the case of impact testing of 3D-printed materials.

Figure 10 illustrates the contrast in use value (impact energy) to production cost ratios for the analyzed parts built for impact testing. This visual representation provides insight into the cost-effectiveness and performance balance of these three materials.

The data in Table 17 reveal a significant increase in the *V_i_/C_p_* ratio for impact testing specimens, ranging from 91.05% to 247.66%. This substantial rise results from the notable improvement in performance outweighing cost increments. Specifically, the production cost for annealed specimens rose moderately, between 9.67% and 12.11%, whereas their impact energy surged significantly, ranging from 114.11% to 283.73%.

**Figure 11:**
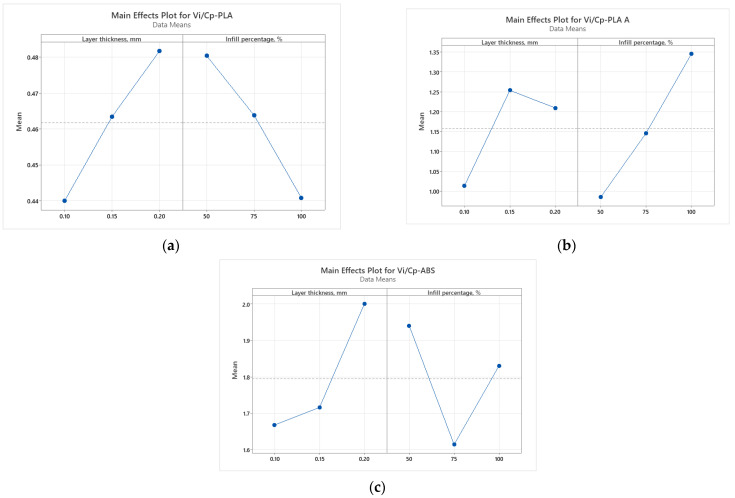
Main effect plots for impact strength: (**a**) PLA; (**b**) PLA A; (**c**) ABS.

**Figure 12:**
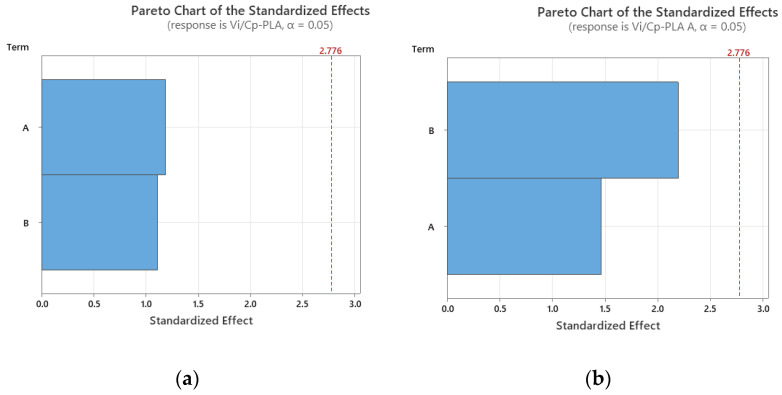

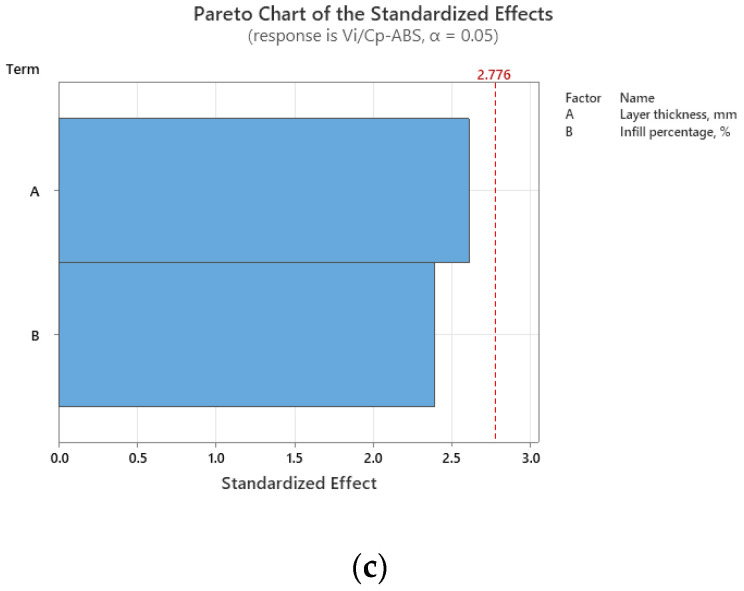
Pareto charts for impact strength: (**a**) PLA; (**b**) PLA A; (**c**) ABS.

In the case of impact strength analysis, both layer thickness and infill percentage influence the *V_i_/C_p_* ratio almost equally, with a slightly higher contribution of layer thickness (for PLA and ABS) and, respectively, infill percentage (for annealed PLA)—see Figure 11 and Figure 12. The infill percentage increase results in increasing the ratio *V_i_/C_p_* in the case of annealed PLA, while for as-built PLA specimens, the ratio decreases with the increase in infill percentage. For both as-built PLA and ABS materials, the increase in the ratio *V_i_/C_p_* is obtained by increasing layer thickness.

#### 3.1.5. Hardness Testing

Table 18 and Table 19 show the results of calculations performed using relations (2…5) to determine the production cost for PLA and ABS samples, respectively, used for hardness testing.

**Figure 13:**
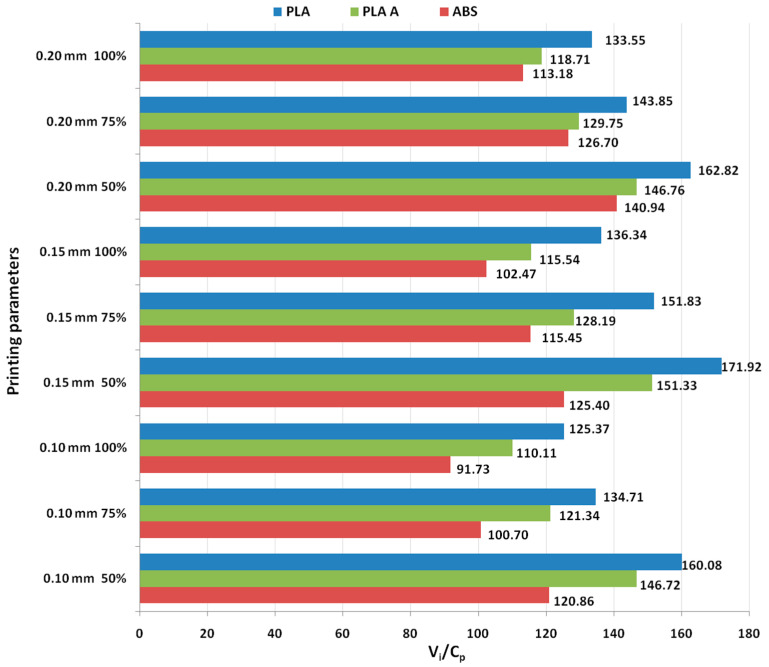
Ratios between use values and production costs in the case of hardness testing of 3D-printed materials.

The *V_i_*/*C_p_* ratio is visually emphasized in Figure 13, aiding in the comprehensive evaluation of these materials based on their hardness characteristics (the use value is represented in this case by Shore D hardness).

The information from Table 20 reveals that, in the context of hardness testing specimens for PLA, the *V_i_/C_p_* ratio exhibited a decline ranging from 8.34% to 15.57%. This reduction is attributed to the fact that the Shore D hardness values of annealed PLA specimens were consistently lower, decreasing by 5.08% to 12.86%. Concurrently, the production cost increased marginally, by 2.79% to 3.74%.

**Figure 14:**
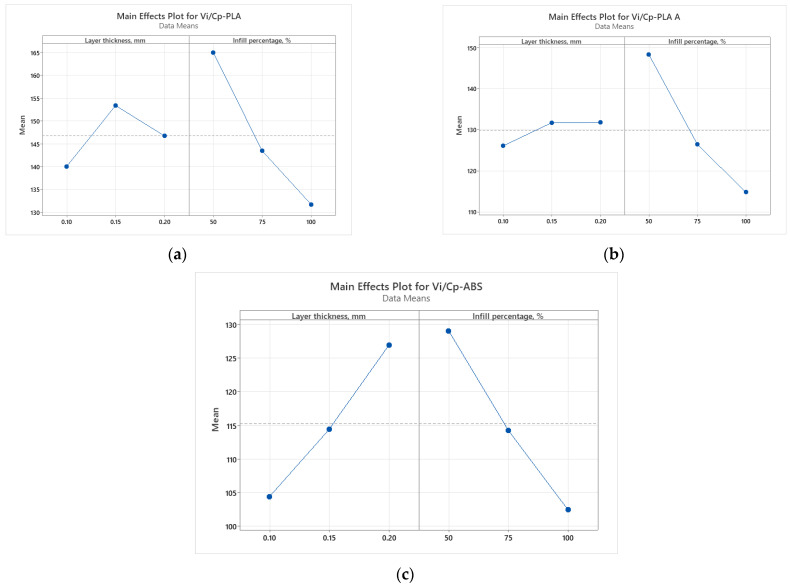
Main effect plots for hardness: (**a**) PLA; (**b**) PLA A; (**c**) ABS.

**Figure 15:**
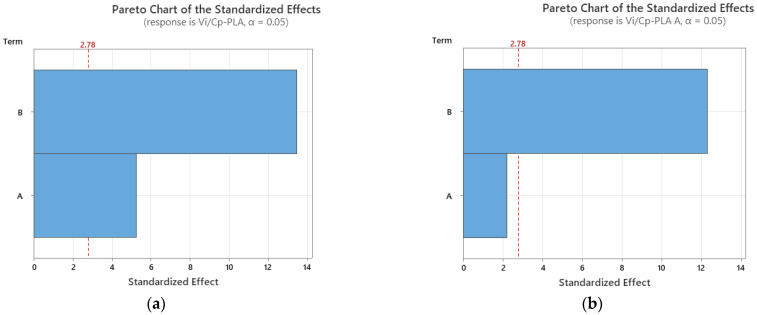

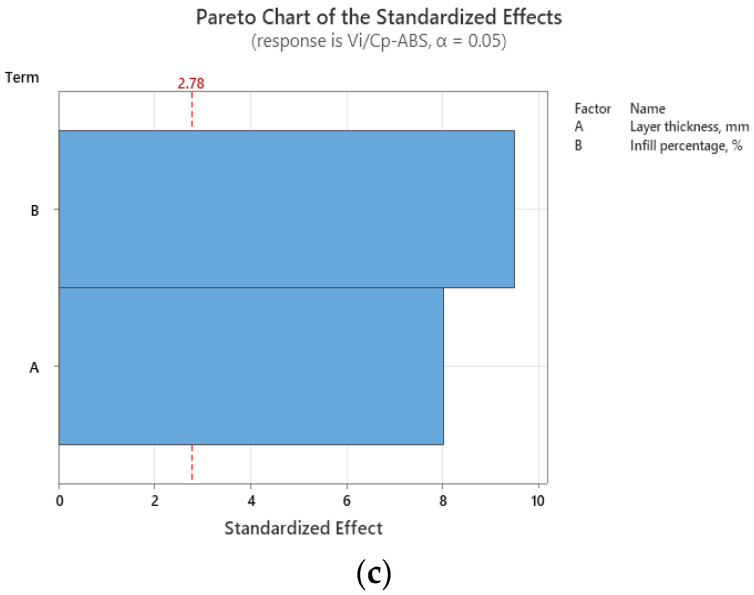
Pareto charts for hardness: (**a**) PLA; (**b**) PLA A; (**c**) ABS.

The main effect plots from Figure 14 and Pareto charts from Figure 15 show that the main factor influencing the ratio *V_i_/C_p_* is the infill percentage, for all the analyzed materials. For PLA the highest ratio is obtained with 0.15 mm layer thickness and 50% infill percentage, for annealed PLA at 0.20 mm layer thickness and 50% infill percentage and for ABS at 0.20 mm layer thickness and 50% infill percentage.

### 3.2. Multi-Response Optimization Using Desirability Function Based on Value Analysis for Enhancing the 3D-Printing Efficiency of PLA and ABS Materials

The objectives of optimization typically involve maximizing, minimizing, or setting target values for a response in order to identify the most favorable processing parameters. In the context of desirability analysis, the objective of the present investigation (for each analyzed material) was to maximize the response desirability, namely the ratio between the use value and production cost (see Table 21), simultaneously for all mechanical characteristics determined previously. Using the process parameters and responses investigated in this study, an optimization approach was applied utilizing the desirability function through Minitab 19 software. Specific optimization goals have been set for the various responses, all of which are aimed maximizing their values. Hence, optimization objectives are established to attain the maximal values for these responses.

Desirability analysis provides values within a range of zero to one, with one indicating the highest level of suitability.

For the case when the importance is the same for each response, the composite desirability *D* is calculated with the formula [3]:

(6) $$D={\left(d_{1}\cdot d_{2}\cdot \ldots \cdot d_{n}\right)}^{1/n}$$

where *n* is the number of responses, *d_i_* represents the desirability for each individual response, calculated (for the case when the goal is to maximize the response desirability) as in [3]:

(7) $$\begin{array}{l} d_{i}=0,\;if\;y_{i}<L_{i}\\ d_{i}=\frac{\left(y_{i}-L_{i}\right)\cdot r_{i}}{\left(T_{i}-L_{i}\right)},\;if\;L_{i}\leq y_{i}\leq T_{i}\\ d_{i}=1,\;if\;y_{i}>T_{i} \end{array}$$

*y_i_*, *T_i_*, *L_i_* represent the predicted value, target value, and lowest value, respectively, of the analyzed response of response. To maintain simplicity, the authors treated all responses as equally significant within the optimization procedure (both weight and importance values were set to one, which means all the variables are equally relevant for the optimization process, as seen in Table 21).

Table 22 displays the rankings assigned to various options concerning the printing parameters for the three materials. The top-ranked option represents the most suitable choice for configuring these parameters to achieve the highest possible ratio between use value and production cost across all responses, as defined in [2,26].

**Figure 16:**
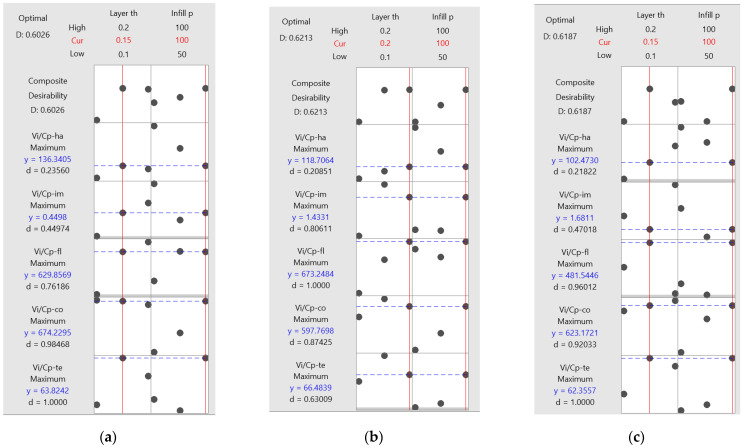
Optimization plots for 3D-printed materials: (**a**) PLA; (**b**) PLA A; (**c**) ABS.

The optimization plot (Figure 16) illustrates how each factor (columns) influences the responses or composite desirability (rows). Vertical red lines on the graph indicate the current factor settings, while the numbers at the top of each column, displayed in red, denote the current factor level settings. The horizontal blue lines and accompanying numbers represent the responses corresponding to the current factor level.

In the case of PLA material, the optimization process yielded specific parameter values, namely a layer thickness of 0.15 mm and a 100% infill percentage, which are visually depicted in red on the optimization plot in Figure 16. Meanwhile, for annealed PLA material, the optimal parameters consist of a 0.20 mm layer thickness and a 100% infill percentage. Lastly, for ABS material, the optimized parameters encompass a 0.15 mm layer thickness and a 100% infill percentage. A similar investigation was performed in [3], and, based on desirability analysis, established the optimal FDM process parameters (layer thickness, speed, and infill percentage) corresponding to minimal values of responses (time, weight of product, and filament length). Regarding the multiobjective optimization, distinct combinations of printing parameters were established in [20] to enhance various aspects such as dimensional accuracy, carbon dioxide emissions, material cost, labor cost, and electricity cost.

## 4. Conclusions

This study introduced a multi-objective optimization approach aimed at identifying the optimal 3D-printing parameters (layer thickness and infill percentage) for achieving the highly efficient production of PLA and ABS 3D-printed parts, based on a complex characterization of their main mechanical properties (tensile strength, compression strength, flexural strength, impact strength, and hardness). The investigation used an innovative solution involving value analysis to establish the optimal printing settings that result in maximizing the ratio between the use value (associated with mechanical characteristics) and the production cost of 3D-printed products. In this way, a technical-economical optimization can be performed.

Based on the obtained results, the following conclusions can be drawn:

✓ For the FDM process under investigation, taking into account the mechanical characteristics (tensile strength, compression strength, flexural strength, impact strength, hardness) and the use value and production cost of specimens made of different materials, the optimum parameters were found.
✓ The ANOVA analysis demonstrated that the infill percentage is the significant factor with statistical influence on the *V_i_/C_p_* ratio in the case of tensile, compression, and hardness testing specimens, while for flexural testing specimens, the layer thickness makes the most important contribution. When analyzing impact strength, both layer thickness and infill percentage exhibit nearly equal influence on the *V_i_/C_p_* ratio. However, for PLA and ABS materials, layer thickness holds a slightly greater impact, while for annealed PLA, the infill percentage has a slightly higher influence.
✓ Annealing has been proven to significantly enhance some mechanical characteristics of PLA 3D-printed parts. In order to obtain a favorable ratio between mechanical performance and production cost, a certain number of samples must be heat-treated simultaneously for an efficient use of energy. The gains in mechanical strength per piece, combined with the efficient use of energy and time during annealing, contribute positively to the cost-effectiveness of the process.
✓ Prospective investigations within this domain necessitate a targeted assessment of value, centered on the specific applications demanded, with a keen focus on enhancing both utility and production-cost efficiency for industry-specific components. To enhance the rigor of future studies, it would be useful to use precise measurements for factors such as energy consumption and labor costs.

## Figures and Tables

**Table 1:** The printing parameters described.

Printing Options for 1 Set of Samples	ABS	PLA
Shell width (mm)	1	1
Infill speed (mm/s)	40	70
Estimated print time (min)	60	46
Estimated filament used (g)	10.60	10.60
Extruder temperature (°C)	240	210
Bed temperature (°C)	110	60
Platform addition	Raft only	Raft only

**Table 2:** Characteristics of filaments from providers’ data sheets.

Materials	Extrusion Temperature (°C)	Bed Temperature (°C)	Density (g/cm^3^)	Tensile Strength (MPa)	Specific Deformation (%)	Charpy Impact Strength (kJ/m^2^)
PLA	210 ± 10	25–60	1.31 ± 0.02	15.5–72	34.5 ± 8.1	5.7 ± 0.4
ABS	210 + 40	110 ± 10	1.10	33.9	4.8	10.5

**Table 3:** Parameters and levels used in DOE analysis.

Parameter	Level		
	1	2	3
Infill percentage, %	50	75	100
Layer thickness, mm	0.10	0.15	0.20

**Table 4:** DOE array.

Experiment no.	Layer Thickness, mm	Infill Percentage, %
1	0.10	50
2		75
3		100
4	0.15	50
5		75
6		100
7	0.20	50
8		75
9		100

**Table 5:** Testing conditions and types of samples used in the experimental investigation.

Mechanical Test	Testing Conditions	Shape and Dimensions of Specimen
Tensile testing	- electro-mechanical machine - force cell of 2.5 kN, - speed of 5 mm/min - with extensometer - ambient temperature 20 °C - humidity 40%	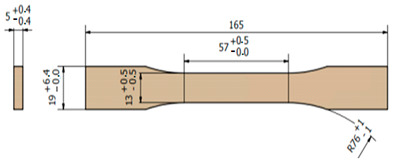
Compression testing	MU 400 Kn universal machine 20 N preload 10 mm/min the speed test ambient temperature 20 °C humidity 40%	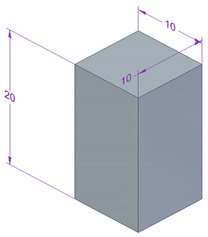
Flexural strength testing	Lloyd LRX Force Tester electro-mechanical - force cell of 2.5 kN, - speed of 5 mm/min - ambient temperature 20 °C humidity 40%	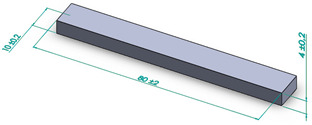
Impact testing	Instron Pendulum Charpy tester Impact velocity 2.9 m/s - ambient temperature 20 °C humidity 40%	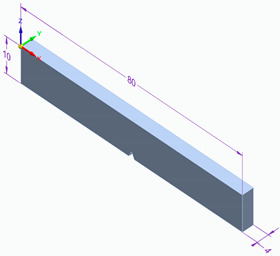
Hardness testing	Shore D type durometer ambient temperature 20 °C humidity 40%	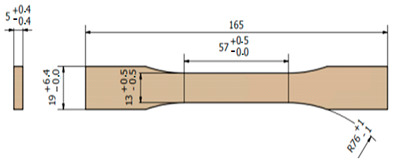

**Table 6:** Cost calculation for PLA samples used for tensile testing.

Experiment No.	*C_mat_*, Euro	*C_e_*, Euro	*C_p_*, Euro	*C_p_^a^*, Euro
1	0.35	0.18	0.52	0.54
2	0.41	0.22	0.62	0.64
3	0.47	0.20	0.67	0.69
4	0.36	0.14	0.50	0.52
5	0.42	0.16	0.58	0.60
6	0.47	0.15	0.63	0.65
7	0.38	0.12	0.50	0.52
8	0.43	0.14	0.56	0.58
9	0.48	0.13	0.61	0.63

**Table 7:** Cost calculation for ABS samples used for tensile testing.

Experiment No.	*C_mat_*, Euro	*C_e_*, Euro	*C_p_*, Euro
1	0.29	0.21	0.50
2	0.34	0.26	0.60
3	0.39	0.26	0.64
4	0.29	0.16	0.45
5	0.34	0.19	0.52
6	0.38	0.18	0.57
7	0.31	0.13	0.44
8	0.35	0.15	0.50
9	0.48	0.13	0.61

**Table 8:** Percentage differences between as-built and annealed samples for PLA tensile specimens.

Experiment No.	Difference, %		
	Ultimate Tensile Strength	*C_p_*	*V_i_/C_p_*
1	25.82	3.56	21.50
2	36.25	2.99	32.29
3	31.04	2.79	27.48
4	15.80	3.71	11.65
5	24.01	3.22	20.15
6	19.91	2.97	16.45
7	15.52	3.74	11.35
8	18.47	3.31	14.67
9	17.00	3.07	13.51

**Table 9:** Cost calculation for PLA samples used for compression testing.

Experiment No.	*C_mat_*, Euro	*C_e_*, Euro	*C_p_*, Euro	*C_p_^a^*, Euro
1	0.05	0.04	0.09	0.11
2	0.06	0.04	0.10	0.12
3	0.07	0.04	0.11	0.12
4	0.05	0.03	0.08	0.10
5	0.06	0.03	0.09	0.11
6	0.07	0.03	0.09	0.11
7	0.05	0.02	0.07	0.09
8	0.06	0.02	0.08	0.10
9	0.07	0.02	0.09	0.11

**Table 10:** Cost calculation for ABS samples used for compression testing.

Experiment No.	*C_mat_*, Euro	*C_e_*, Euro	*C_p_*, Euro
1	0.04	0.04	0.08
2	0.05	0.05	0.10
3	0.05	0.05	0.10
4	0.04	0.03	0.07
5	0.05	0.03	0.08
6	0.05	0.03	0.09
7	0.04	0.02	0.06
8	0.05	0.03	0.07
9	0.05	0.03	0.08

**Table 11:** Percentage differences between as-built and annealed samples for PLA compression specimens.

Experiment no.	Difference, %		
	Compressive Stress	*C_p_*	*V_i_/C_p_*
1	5.78	20.84	−14.24
2	9.36	18.04	−7.93
3	−5.88	17.74	−25.10
4	13.84	23.33	−8.34
5	13.60	20.57	−6.14
6	13.02	19.98	−6.16
7	13.07	25.52	−11.01
8	9.72	22.25	−11.42
9	10.29	20.57	−9.33

**Table 12:** Cost calculation for PLA samples used for flexural strength testing.

Experiment No.	*C_mat_*, Euro	*C_e_*, Euro	*C_p_*, Euro	*C_p_^a^_,_* Euro
1	0.12	0.05	0.17	0.19
2	0.13	0.06	0.19	0.21
3	0.13	0.06	0.19	0.21
4	0.12	0.04	0.17	0.18
5	0.13	0.05	0.18	0.19
6	0.13	0.05	0.18	0.20
7	0.13	0.04	0.16	0.18
8	0.13	0.04	0.17	0.19
9	0.13	0.04	0.17	0.19

**Table 13:** Cost calculation for ABS samples used for flexural strength testing.

Experiment No.	*C_mat_*, Euro	*C_e_*, Euro	*C_p_*, Euro
1	0.09	0.06	0.15
2	0.10	0.08	0.17
3	0.11	0.08	0.18
4	0.09	0.05	0.14
5	0.10	0.06	0.15
6	0.11	0.06	0.16
7	0.09	0.04	0.13
8	0.10	0.05	0.14
9	0.11	0.05	0.15

**Table 14:** Percentage differences between as-built and annealed samples for PLA flexural specimens.

Experiment No.	Difference, %		
	Flexural Strength	*C_p_*	*V_i_/C_p_*
1	9.88	10.93	−0.95
2	11.66	9.91	1.59
3	8.10	9.57	−1.34
4	16.37	11.29	4.57
5	2.12	10.61	−7.68
6	10.20	10.36	−0.14
7	16.72	11.55	4.63
8	8.05	11.17	−2.81
9	15.23	10.89	3.92

**Table 15:** Cost calculation for PLA samples used for impact testing.

Experiment No.	*C_mat_*, Euro	*C_e_*, Euro	*C_p_*, Euro	*C_p_^a^_,_* Euro
1	0.11	0.06	0.16	0.18
2	0.12	0.07	0.19	0.21
3	0.13	0.06	0.19	0.21
4	0.11	0.04	0.15	0.17
5	0.12	0.05	0.17	0.19
6	0.13	0.05	0.18	0.20
7	0.12	0.04	0.15	0.17
8	0.13	0.04	0.17	0.18
9	0.14	0.04	0.18	0.19

**Table 16:** Cost calculation for ABS samples used for impact testing.

Experiment No.	*C_mat_*, Euro	*C_e_*, Euro	*C_p_*, Euro
1	0.09	0.06	0.15
2	0.10	0.08	0.18
3	0.11	0.08	0.19
4	0.09	0.05	0.14
5	0.10	0.06	0.16
6	0.11	0.06	0.17
7	0.09	0.04	0.13
8	0.10	0.05	0.15
9	0.11	0.05	0.16

**Table 17:** Percentage differences between as-built and annealed samples for PLA impact testing specimens.

Experiment No.	Difference, %		
	Impact Energy	*C_p_*	*V_i_/C_p_*
1	125.08	11.43	101.99
2	157.63	10.02	134.17
3	181.30	9.67	156.49
4	114.11	12.07	91.05
5	213.64	10.84	182.96
6	283.73	10.37	247.66
7	148.98	12.11	122.09
8	152.84	11.23	127.32
9	238.83	10.63	206.27

**Table 18:** Cost calculation for PLA samples used for hardness testing.

Experiment No.	*C_mat_*, Euro	*C_e_*, Euro	*C_p_*, Euro	*C_p_^a^_,_* Euro
1	0.35	0.18	0.52	0.54
2	0.41	0.22	0.62	0.64
3	0.05	0.20	0.25	0.69
4	0.36	0.14	0.50	0.52
5	0.42	0.16	0.58	0.60
6	0.47	0.15	0.63	0.65
7	0.38	0.12	0.50	0.52
8	0.43	0.14	0.56	0.58
9	0.48	0.13	0.61	0.63

**Table 19:** Cost calculation for ABS samples used for hardness testing.

Experiment No.	*C_mat_*, Euro	*C_e_*, Euro	*C_p_*, Euro
1	0.29	0.21	0.50
2	0.34	0.26	0.60
3	0.39	0.26	0.64
4	0.29	0.16	0.45
5	0.34	0.19	0.52
6	0.38	0.18	0.57
7	0.31	0.13	0.44
8	0.35	0.15	0.50
9	0.39	0.15	0.55

**Table 20:** Percentage differences between as-built and annealed samples for PLA hardness testing specimens.

Experiment No.	Difference, %		
	Shore D Hardness	*C_p_*	*V_i_/C_p_*
1	−5.08	3.56	−8.34
2	−7.23	2.99	−9.92
3	−9.73	2.79	−12.17
4	−8.71	3.71	−11.98
5	−12.86	3.22	−15.57
6	−12.73	2.97	−15.25
7	−6.49	3.74	−9.86
8	−6.82	3.31	−9.80
9	−8.38	3.07	−11.11

**Table 21:** Optimization Goals for analyzed materials.

Response, *V_i_/C_p_*	Goal	Lower			Target			Weight	Importance
		PLA	PLA A	ABS	PLA	PLA A	ABS		
Hardness, [Shore D hardness/Euro]	Maximum	125.37	110.111	91.73	171.92	151.33	140.94	1	1
Impact, [J/Euro]		0.40	0.889	1.32	0.50	1.56	2.08		
Flexural, [MPa/Euro]		553.49	546.066	363.17	653.72	673.24	486.46		
Compression, [MPa/Euro]		386.33	338.179	268.93	678.70	635.11	653.83		
Tensile, [MPa/Euro]		42.26	53.132	27.06	63.82	74.32	62.35		

**Table 22:** Composite Desirability and Ranks.

Printing Parameters		Material					
		PLA		PLA A		ABS	
Layer Thickness, mm	Infill Percentage, %	Composite Desirability	Rank	Composite Desirability	Rank	Composite Desirability	Rank
0.10	50	0.0092	7	0.0000	9	0.0000	7
	75	0.0000	9	0.2664	5	0.0252	6
	100	0.0000	9	0.0002	7	0.0000	7
0.15	50	0.3302	6	0.2528	6	0.3840	4
	75	0.4318	4	0.3681	3	0.0000	7
	100	0.6026	1 *	0.6121	2	0.6187	1 *
0.20	50	0.4298	5	0.0000	9	0.5058	2
	75	0.5093	3	0.3209	4	0.4674	3
	100	0.5909	2	0.6213	1 *	0.3690	5

* the highlighted lines correspond to the optimal values (having Rank 1) of printing parameters.

## Data Availability

Data are contained within the article.
